# Supplementary material for: A Survey-Based Analysis of Injuries to Horses Associated with Transport by Road in New Zealand
Source: Animals (Basel). 2022 Jan 21;12(3):259. doi: 10.3390/ani12030259 (PMC8833432; doi:10.3390/ani12030259)
Supplement: Supplementary file 1 [file animals-12-00259-s001.zip › animals-1539755-supplementary.pdf]

Table S1: Variables with a Wald test *p*-value ≥ 0.25 that were not eligible for initial inclusion in the multivariate logistic regression model based on the results of univariate logistic regression analyses of associations between horse injury in transit and industry sector, vehicle safety design features, and horse protective equipment used to protect horses in transit, and transport-related behavioural problems.

**Variable Name**	**Category**	**Est.**[1]	**SE**[2]	**OR**[3]	**95% CI**[4]	***p***[5]
Vehicle type	Float/trailer - angle load	-0.13	0.37	0.99	0.47-2.06	0.834
	Float/trailer - straight	-0.09	0.29	0.92	0.53-1.67	
	Commercial truck	0.19	0.42	1.20	0.52-2.72	
	Large truck - > 3 horses	0.16	0.32	1.18	0.63-2.26	
	Small truck - 2 to 3 horses	Ref				
Sedation used for transport	Yes	0.14	0.20	1.15	0.77-1.69	0.489
	No	Ref				
Horse specifically trained for transport	Yes	-0.10	0.21	0.91	0.60-1.40	0.647
	No	Ref				
Neck rug used	Yes	-0.30	0.36	0.74	0.38-1.59	0.422
	No	Ref				
Body rug used	Yes	0.12	0.20	1.13	0.77-1.68	0.530
	No	Ref				
Leg bandage/boots used	Yes	0.18	0.18	1.20	0.84-1.72	0.319
	No	Ref				
Head restraint in the moving vehicle	Crosstied	0.69	0.49	2.00	0.74-5.13	0.574
	Elastic Bungee cord	-0.12	0.45	0.88	0.35-2.09	
	Tied on a long rope	-0.19	0.34	0.83	0.43-1.64	
	Tied on a short rope	-0.09	0.29	0.92	0.53-1.67	
	Not restrained	Ref				
Padded walls in the vehicle	Yes	-0.01	0.18	0.99	0.70-1.40	0.956
	No	Ref				
Padded chest bar in the vehicle	Yes	-0.12	0.18	0.89	0.62-1.26	0.509
	No	Ref				
Padded bum (rear) bar in the vehicle	Yes	-0.02	0.18	0.98	0.68-1.40	0.925
	No	Ref				
Padded partition in the vehicle	Yes	0.12	0.18	1.12	0.79-1.60	0.513
	No	Ref				
Partition between horses extends to the floor	Yes	0.17	0.29	1.12	0.65-2.04	0.555
	No	Ref				

[1]Coefficient estimate; [2]Standard error; [3]Odds ratio; [4]95% Confidence interval; [5]Wald test *p*-value.
